# Repeated PSMA-targeting radioligand therapy of metastatic prostate cancer with ^131^I-MIP-1095

**DOI:** 10.1007/s00259-017-3665-9

**Published:** 2017-03-09

**Authors:** Ali Afshar-Oromieh, Uwe Haberkorn, Christian Zechmann, Thomas Armor, Walter Mier, Fabian Spohn, Nils Debus, Tim Holland-Letz, John Babich, Clemens Kratochwil

**Affiliations:** 1 Department of Nuclear Medicine, Heidelberg University Hospital, INF 400, 69120 Heidelberg, Germany; 2 Clinical Cooperation Unit Nuclear Medicine, German Cancer Research Center, Heidelberg, Germany; 3 Progenics Pharmaceuticals, Inc., New York, NY USA; 4 Department of Biostatistics, German Cancer Research Center, Heidelberg, Germany; 5 Division of Radiopharmaceutical Sciences, Department of Radiology, Weill Cornell Medicine, New York, NY USA; 6 Citigroup Biomedical Imaging Center, Weill Cornell Medicine, New York, NY 10021 USA; 7 Meyer Cancer Center, Weill Cornell Medicine, New York, NY 10021 USA

**Keywords:** Prostate cancer, PSMA, Prostate-specific membrane antigen, Endoradiotherapy

## Abstract

**Purpose:**

Prostate-specific membrane antigen (PSMA)-targeting radioligand therapy (RLT) was introduced in 2011. The first report described the antitumor and side effects of a single dose. The aim of this analysis was to evaluate toxicity and antitumor activity after single and repetitive therapies.

**Methods:**

Thirty-four men with metastatic castration-resistant prostate cancer received PSMA-RLT with ^131^I-MIP-1095. Twenty-three patients received a second, and three patients a third dose, timed at PSA progression after an initial response to the preceding therapy. The applied doses were separated in three groups: <3.5, 3.5–5.0 and >5.0 GBq. Antitumor and side-effects were analyzed by blood samples and other clinical data. Follow-up was conducted for up to 5 years.

**Results:**

The best therapeutic effect was achieved by the first therapy. A PSA decline of ≥50% was achieved in 70.6% of the patients. The second and third therapies were significantly less effective. There was neither an association between the applied activity and PSA response or the time-to-progression. Hematologic toxicities were less prevalent but presented in a higher percentage of patients with increasing number of therapies. After hematologic toxicities, xerostomia was the second most frequent side effect and presented more often and with higher intensity after the second or third therapy.

**Conclusion:**

The first dose of RLT with ^131^I-MIP-1095 presented with low side effects and could significantly reduce the tumor burden in a majority of patients. The second and third therapies were less effective and presented with more frequent and more intense side effects, especially hematologic toxicities and xerostomia.

**Electronic supplementary material:**

The online version of this article (doi:10.1007/s00259-017-3665-9) contains supplementary material, which is available to authorized users.

## Introduction

The clinical translation of ^99m^Tc-, ^18^F- and ^68^Ga-labeled prostate specific membrane antigen (PSMA)-targeting tracers for single-photon emission tomography (SPECT) or positron emission tomography (PET) are considered a significant step forward for the diagnostics of prostate cancer (PCa) [1–5]. The very first small-molecule PSMA ligands that presented convincing results in men were ^123^I-MIP-1072 and ^123^I-MIP-1095 [6]; which were described in the literature in 2009 [7]. However, these pioneering ligands were under-recognized at their introduction. One reason might be that the medium-energy cyclotron-produced ^123^I is more expensive then compact cyclotron-produced ^18^F or generator nuclides such as ^99m^Tc or ^68^Ga. On-site labeling with radioiodine can also be more complex than labeling of chelator-containing ligands.

Since internalization occurs after binding of antibodies and small-molecule ligands to PSMA, these molecules may also be good candidates for endoradiotherapy [8]. First therapy was done with the monoclonal antibody J591. In phase 1 and 2 clinical trials, J591 was radiolabeled with ^90^Y or ^177^Lu leading to promising early results [9–11]. However, monoclonal antibodies are large molecules which show poor permeability in solid tumors and slow clearance from the circulation. Due to their specific tumor targeting and faster pharmacokinetics in comparison to full-length antibodies, small-molecule PSMA ligands were also considered promising for systemic radioligand therapy (RLT) of metastatic prostate cancer. DOTA or DOTAG -chelator-containing ligands can easily be labeled with a variety of different diagnostic or therapeutic radionuclides, making them favorable candidates for clinical application. Finally, such compounds became available in 2013 and first clinical results were now published in 2016 [12–21]. However, the first PSMA RLTs based on a radioiodine-labeled compound were already conducted between July 2011 and June 2012 [22]. After PET-based dosimetry with ^124^I-MIP-1095, 28 patients received one single treatment with ^131^I-MIP-1095 and were followed until first PSA progression [22]. An additional eight patients received their first cycle of ^131^I-MIP-1095 afterwards, before PSMA RLT was switched to ^177^Lu-PSMA ligands.

Here, we report our long-term follow-up of 34 patients with a special focus on repeated ^131^I-MIP-1095 therapies, which were not timed at fixed intervals but were only scheduled after progression of disease.

## Materials and methods

### Patients

Between July 2011 and October 2013, 36 men with progressive metastatic castration-resistant prostate cancer (mCRPC) were referred to PSMA RLT after having received all approved therapies available at that time.

A PSMA-positive tumor phenotype was demonstrated with ^68^Ga-PSMA-11 PET/CT. The characteristics of all 36 patients who received at least one therapy with ^131^I-MIP-1095 are given in Table 1. Out of these, two patients were lost for follow-up. Twenty-three of the remaining patients received a second and three patients received a third therapy. Overall, 60 treatments were applied in the 34 evaluable patients. The second and third therapeutic activity was applied in case of PSA progression following an initial decline after the first therapy (supplementary figure 1).

**Table 1:** Patients’ characteristics

	1st Therapy	2nd Therapy	3rd Therapy
Dose (GBq)	4.2 ± 1.4 (1.9–7.2)	3.2 ± 1.2 (2.0–6.4)	2.4 ± 1.0 (1.5–3.5)
Time to PSA progression (d)	116 ± 141 (18–735)	62 ± 28 (28–117)	42*
PSA baseline (ng/ml)	261 (1.1–2109.0)	212 (0.3–1497.0)	480 (125.0–878.0)
	Chemotherapy prior to PSMA therapy	CRPC	Age (mean, range)
	14/34 (44%)	All patients	68 (49–81)
Types of PCa			
Local PCa (3 patients)	Lymph node metastases (18 patients)	Visceral metastases (3 patients)	Bone metastases (28 patients)

^*^Only one patient presented with PSA decline after the third cycle; all others presented with continuous progressive disease despite RLT

The follow-up until first PSA progression of 28 patients receiving one single therapy has previously been published [22].

Depending on the efficiency of radiolabeling, patients received an average radioactivity of 4.2 ± 1.4 GBq (1.9–7.2; median 3.7) for the first therapy. The activities of the second and third therapies were also depending on labeling yields. However, if blood cell count was not completely recovered, the responsible physician could indicate individual dose reductions. For the second therapy, we applied 3.2 ± 1.2 GBq (2.0–6.4; median 2.7) and for the third therapy, 2.4 ± 1.0 GBq (1.5–3.5; 2.2). In order to evaluate possible association of side effects with therapy doses, the applied doses were separated in three groups: <3.5 GBq (group 1), 3.5–5.0 GBq (group 2) and >5.0 GBq (group 3).

On the first (prior to radioactivity administration) and on the last day of the hospitalization, blood samples were collected from all patients for the measurement of the following parameters: hematology, electrolytes, glutamic oxaloacetic transaminase, glutamate-pyruvate transaminase, gamma-glutamyl transferase, alkaline phosphatase, bilirubin, urea, creatinine, glomerular filtration rate, prostate-specific antigen (PSA), thyroid-stimulating hormone, free triiodothyronine and free thyroxine.

In order to reduce possible thyroid uptake of free radioiodine, 60 drops of sodium perchlorate (Irenat®, Bayer, Berlin, Germany) were given p.o. ca. ½ h prior to therapy and 3 × 20 drops in the next 14 days after therapy administration. Also, prior to therapy, the patients received 1000–2000 mL of 0.9% NaCl solution over 1 day. The therapy solution (described below) was administered by intravenous infusion over 20–30 min.

Post administration, patients were treated as in-patients on the nuclear medicine therapy ward for 5–8 days according to German radiation protection laws. The vital parameters as well as side effects or adverse effects were recorded during the whole hospitalization time. In an attempt to to reduce therapy-induced damage of the salivary glands, the patients received five times per day lemon juice and ice packs during the first day to reduce organ perfusion over the parotids and submandibular glands for the period of their hospitalization.

Whole-body scintigraphy was acquired at the last day of the hospitalization. The patients were then followed-up further for side effects and blood parameters taken every 2 weeks over a period of 10 weeks (the thyroid parameters were controlled monthly by blood tests). After the 10th week, the frequency of the blood analyses was at the discretion of the treating oncologists. Any further (long-term) available information about the relevant medical history of the patients was collected until the death of the patients. At the time this manuscript was submitted, four patients were still living while all others have passed away.

Hematologic toxicities were analyzed according to common terminology criteria (CTC) of the World Health Organization. Xerostomia grades were as follows: grade 0 (no toxicity), grade 1 (measurable in sctintigraphy only), grade 2 (noticeable for patients, but no treatment required), grade 3 (treatment required), grade 4 (significant impairment) and grade 5 (percutaneous endoscopic gastrostomy required). Dysgeusia, xerophthalmia and fatigue syndrome were recorded as present or absent, including their duration. Bone pain was recorded as present or absent including, its change after the therapy.

In case of biochemical relapse of PCa, the patients were re-evaluated for an additional therapy with ^131^I-MIP-1095. Requirements for this additional therapy were a sufficient bone marrow reserve (leucocytes ≥3000/μl blood; platelets ≥75,000/μl blood) and a response to the prior therapy shown by PSA decline and/or morphological response demonstrated by computed tomography (CT), magnetic resonance imaging (MRI) or positron emission Tomography (PET) with ^68^Ga-PSMA-11.

### Radiopharmaceutical

The radioiodinated compound ^131^I-MIP-1095 was prepared as described previously [22]. Briefly, ^131^I-MIP-1095 was prepared by iododestannylation of the trimethylstannyl precursor (S)-ditert-butyl2-(3-((S)-1-tert-butoxy-1-oxo-6-(3-(4-(trimethylstannyl)phenyl)ureido)-hexane-2-yl)ureido)pentanedioate, to form (S)-2-(3-((S)-1-carboxy-5-(3-(4 [131I]iodophenyl)ureido)-pentyl)ureido)pentanedioic acid. [131I]NaI (approx. 7.4 GBq, GE Healthcare) was reacted with 100 μL of a 250-μg/mL solution of (S)-di-tert-butyl2-(3-((S)-1-tert-butoxy-1-oxo-6-(3-(4-(trimethylstannyl)phenyl)ureido)hexane-2-yl)ureido)pentanedioate in ethanol and 50 μL of a freshly prepared solution of 0.15 mL of 30% hydrogen peroxide in 0.85 mL of acetic acid. The reaction mixture was diluted after 5 min with 1.5 mL of water and loaded onto a SOLA cartridge. The cartridge was washed with 2 mL of water to remove unreacted radioiodide and inorganic and organic salts and dried by a stream of nitrogen. The cartridge was eluted using 500 μl of neat trifluoroacetic acid (TFA) and incubated at room temperature (RT) for 7 min. Upon dilution with 5 ml of H_2_O, the product was loaded onto a Bond Elut cartridge and washed with 5 ml of 20% ethanol in water; the product was eluted using 1 ml of ethanol, neutralized with phosphate buffer and sterile-filtered.

### Statistical analysis

For statistical analysis, SigmaPlot version 12 (Systat Software Inc., Chicago, IL, USA) was used. Patients were classified into three groups (as mentioned above) according to the administered therapeutic doses. Differences between these three groups with regard to PSA response, decline of leucocytes, decline of platelets and time to PSA progression were assessed for statistical significance using Kruskal–Wallis tests. In all cases, a *p* value of <0.05 was considered statistically significant.

## Results

### Therapy efficacy

**Fig. 1:**
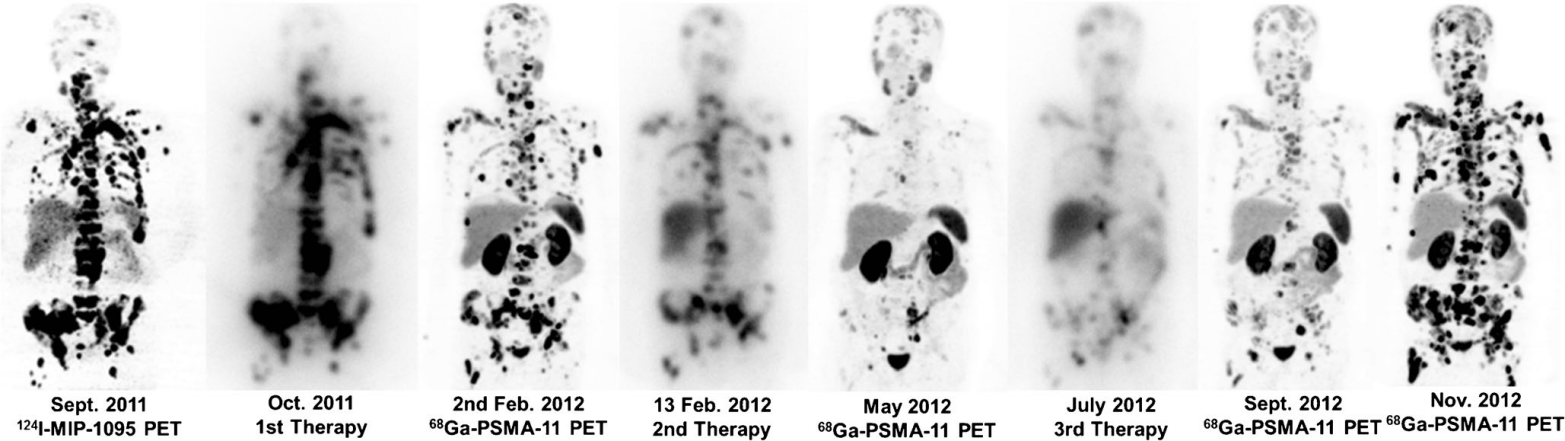
Patient no. 2 received three therapies with ^131^I-MIP-1095. Staging before and after each therapy was conducted with PSMA ligand PET/CT (^124^I-MIP-1095 and ^68^Ga-PSMA-11). The first two therapies reduced the tumor burden. However, the third therapy didn’t show a sufficient effect. The last two pictures on the right side show the rapid progress between September and November. The images of the PET scans show the maximum intensity projections, those of the therapies show the geometric mean of the gamma-ray co-emission which enables imaging during therapy

**Fig. 2:**
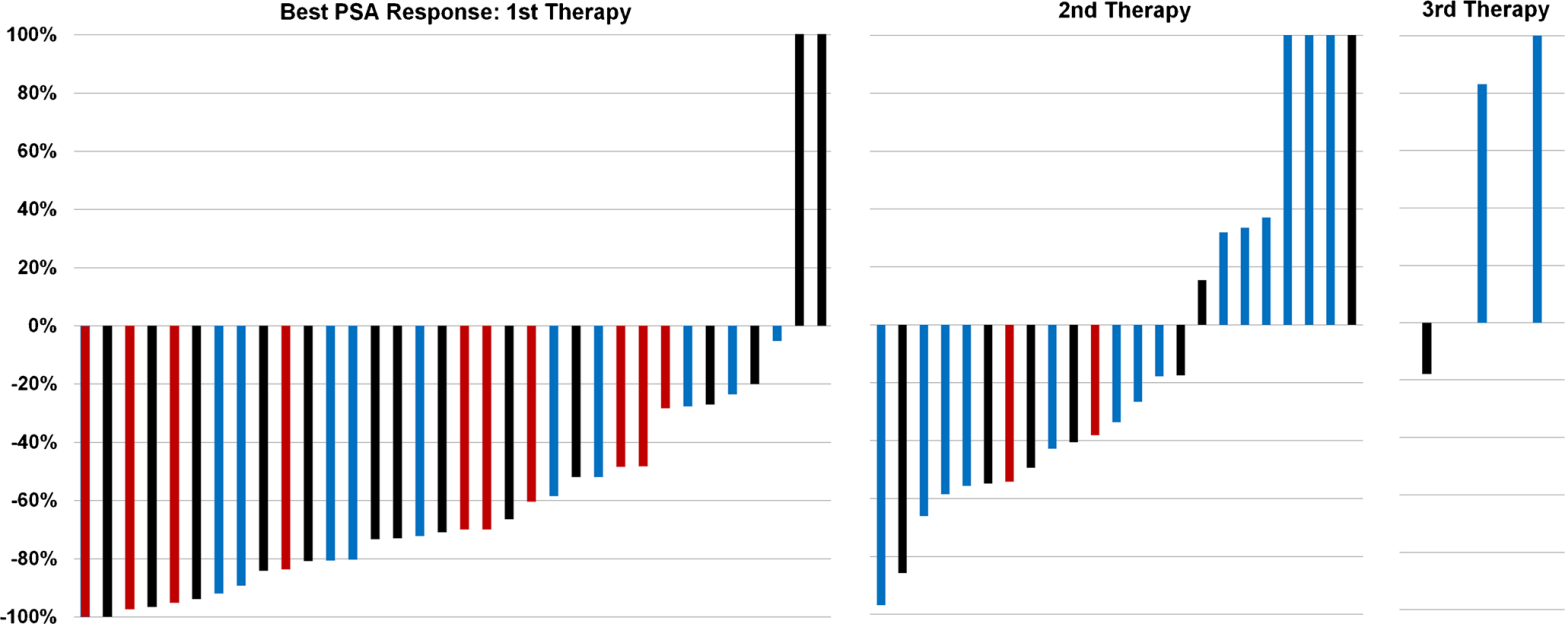
Best PSA response after the first, second and third therapies with ^131^I-MIP-1095. As demonstrated by this figure, the second and third therapies were significantly less effective compared to the first therapy. There was no association between the applied activity and the PSA response (first therapy: *p* = 0.70; second therapy: *p* = 0.74). *Blue bars*: patient group 1 (<3.5 GBq applied activity); *black bars*: patient group 2 (3.5–5.0 GBq applied activity); *red bars*: patient group 3 (>5 GBq applied activity)

**Fig. 3:**
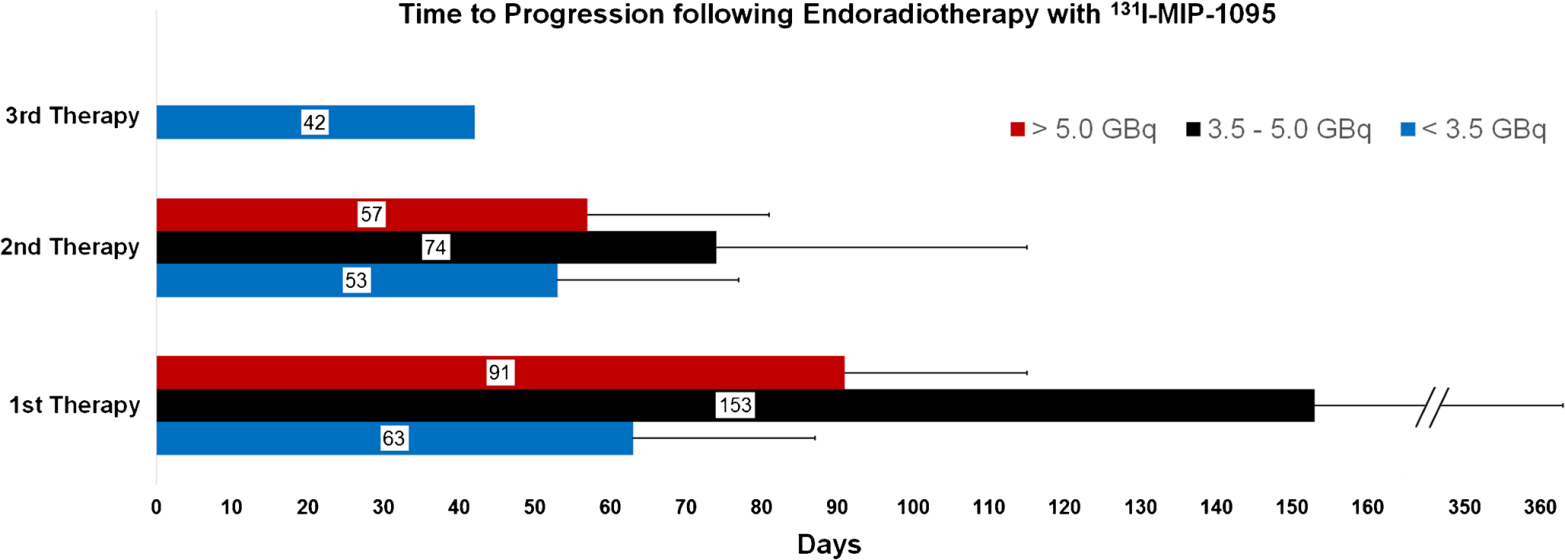
Average time to PSA progression following 1–3 RLT with ^131^I-MIP-1095. There was no association between applied radioactivity and the time to progression (first therapy: *p* = 0.19; second therapy: *p* = 0.61)

Figure 1 represents a case of disease progression following therapy response. The best PSA responses to the RLTs are listed in Fig. 2. Different levels of PSA response following the first, second and third therapies are listed in Table 2. With regard to the whole patient cohort, mean time to PSA progression (TTP) was 116 ± 141 days (range 18–735; median 75) after the first therapeutic administration, 60 ± 28 days (range 28–117; median 50) after the second and 42 days (*n* = 1) after the third PSMA RLT with ^131^I-MIP-1095. Dose-related PSA response and time to progression after the endoradiotherapies are listed in Table 3 and Fig. 3.

**Table 2:** PSA responses to the first, second and third therapies with ^131^I-MIP-1095

	Any PSA response	≥25% PSA decline	≥50% PSA decline	≥75% PSA decline
1st therapy	*n* = 32 (94.1%)	*n* = 29 (85.3%)	*n* = 24 (70.6%)	*n* = 13 (38.2%)
2nd therapy	*n* = 15 (65.2%)	*n* = 13 (56.5%)	*n* = 7 (30.4%)	*n* = 2 (8.7%)
3rd therapy	*n* = 1 (33.3%)	*n* = 0	*n* = 0	*n* = 0

**Table 3:** Dose-related PSA response and time to progression following endoradiotherapy with ^131^I-MIP-1095

	Group 1 (<3.5 GBq)	Group 2 (3.5–5.0 GBq)	Group 3 (>5.0 GBq)
1st Therapy	(*n* = 10)	(*n* = 14)	(*n* = 10)
Best PSA response ≥25%	100%	79%	100%
Best PSA response ≥50%	60%	64%	70%
Best PSA response ≥75%	40%	36%	40%
Any PSA response	100%	86%	100%
TTP (PSA; days)^*^	63 ± 24 (28–90; 75)	153 ± 210 (18–735; 65)	91 ± 24 (28–91; 56)
2nd Therapy	(*n* = 15)	(*n* = 5)	(*n* = 3)
Best PSA response ≥25%	50%	57%	100%
Best PSA response ≥50%	29%	57%	50%
Best PSA response ≥75%	7%	14%	0%
Any PSA response	57%	71%	100%
TTP (PSA; days)^*^	53 ± 24 (28–91; 50)	74 ± 41 (29–117; 74)	57 ± 24 (28–91; 56)
3rd Therapy	(*n* = 2)	(*n* = 1)	
Best PSA response ≥25%	0%	100%	–
Best PSA response ≥50%	0%	0%	–
Best PSA response ≥75%	0%	0%	–
Any PSA response	0%	100%	–
TTP (PSA; days)^*^	42^∼^	–	–

^*^TTP: time to progression of PSA in days. ^∼^Only one patient presented with PSA decline after the third cycle; all others presented with continuous progressive disease despite RLT

As visible by the mentioned figure and tables, the second and third therapies were significantly less effective compared to the first therapy. There was neither an association between the applied activity and PSA response (first therapy: *p* = 0.70; second therapy: *p* = 0.74) nor between the applied activity and the time to progression (first therapy: *p* = 0.19; second therapy: *p* = 0.61).

### Safety and side effects

No acute toxicity or side effects were observed in any patient during their hospitalization time (5–8 days) except one patient who reported dysgeusia after the second therapy. The dysgeusia went on for 2 weeks.

**Fig. 4:**
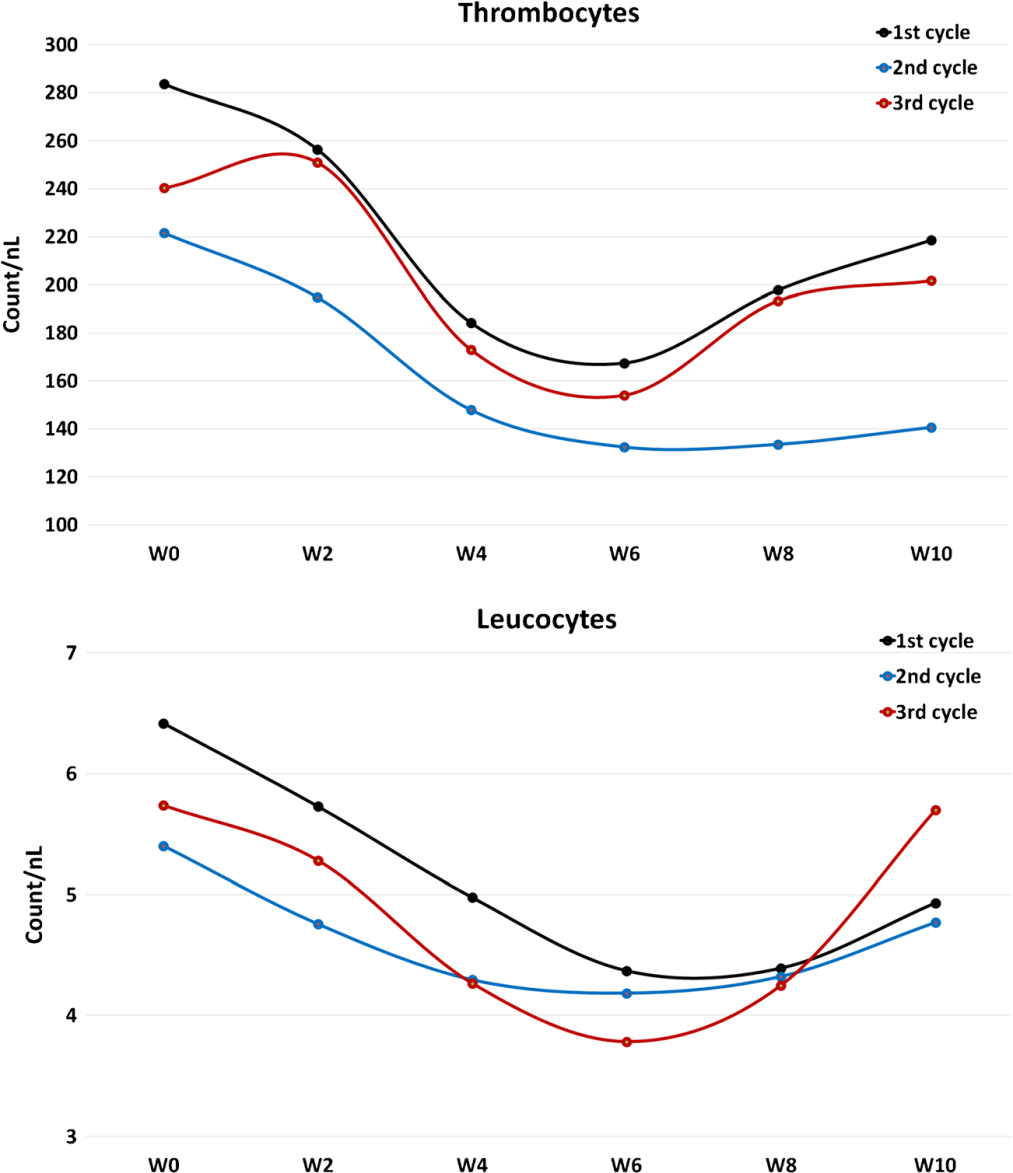
Clinically relevant hematologic toxicity was observed with regard to platelets and leucocytes. W0-W10: average values of the pretherapeutical state (W0) to 10 weeks after therapy administration (W10)

A measurable decline of blood cells within the first 10 weeks after each therapy was observed for leucocytes and platelets only (Fig. 4). However, according to the WHO CTC, no clinically relevant hematologic toxicities were observed in the majority of the patients (Table 4). CTC grade 1, 2 and 3 hematologic toxicities were less prevalent but presented in a higher percentage of patients with increasing number of therapies (Table 4).

**Table 4:** Side effects and effects on bone pain of endoradiotherapy with ^131^I-MIP-1095

	1st Therapy	2nd Therapy	3rd Therapy
Thrombopenia	No relevant toxicity in 28 patients (82.4%)	No relevant toxicity in 15 patients (65.2%)	No relevant toxicity in 2 patients (66.6%)
(CTC Grade)	Grade 1 in 3 patients (8.58%)	Grade 1 in 2 patients (8.7%)	–
	Grade 2 in 1 patients (2.9%)	Grade 2 in 3 patients (13.0%)	–
	Grade 3 in 2 patients (5.9%)	Grade 3 in 2 patients (8.7%)	–
	–	–	Grade 4 in 1 patient (33.3%)
	–	Grade 1 → Grade 3 in 1 patient (4.3%)	–
Leukopenia	No relevant toxicity in 20 patients (58.8%)	No relevant toxicity in 11 patients (47.8%)	No relevant toxicity in 1 patient (33.3%)
	Grade 1 in 8 patients (23.5%)	Grade 1 in 6 patients (26.0%)	Grade 1 in 1 patient (33.3%)
	Grade 2 in 5 patients (14.7%)	Grade 2 in 4 patients (17.4%)	Grade 2 in 1 patient (33.3%)
	Grade 3 in 1 patient (2.9%)	–	–
	–	Grade 1 → grade 2 in 2 patient (8.7%)	–
Xerostonia	Grade 0 in 4 patients (11.8%)	Grade 0 in 0 patient (0.0%)	Grade 0 in 0 patients (0.0%)
	Grade 1 in 24 patients (70.6%)	Grade 1 in 14 patients (60.9%)	Grade 1 in 2 patients (66.6%)
	Grade 2 in 6 patients (17.6%)	Grade 2 in 6 patients (26.1%)	Grade 2 in 0 patients (0.0%)
	Grade 3 in 0 patients (0.0%)	Grade 3 in 3 patients (13.0%)	Grade 3 in 1 patient (33.3%)
Dysgeusia	None	1 Patient (4.3%)	None
Xerophthalmia	None	1 Patient (4.3%)	None
Fatigue	2 Patients (5.9%)	2 Patients (8.7%)	1 Patient (33.3%)
Bone pain	Pain prior to therapy: 16 patients (47%)	Pain prior to therapy: 11 patients (47.8%)	Pain prior to therapy: 2 patients (66.6%)
	Reduction after therapy: 15 patients (93.8%)	Reduction after therapy: 3 patients (27.3%)	Reduction after therapy: 0 patients (0%)
	Uncharged intensity after therapy: 1 patient (6.2%)	Uncharged intensity after therapy: 8 patients (72.7%)	Uncharged intensity after therapy: 2 patients (100%)

No significant value alterations were observed for glutamic oxaloacetic transaminase, glutamate-pyruvate transaminase, gamma-glutamyl transferase, alkaline phosphatase, bilirubin, urea, creatinine, glomerular filtration rate, thyroid-stimulating hormone, free triiodothyronine and free thyroxine (data not shown).

No association was found between the three different dose groups (<3.5; 3.5–5.0 and >5.0 GBq) and best PSA response, decline of leucocytes, decline of platelets and time to PSA progression after the therapies. The *p* values for the first therapy were as follows: 0.70 for best PSA response; 0.26 for decline of leucocytes, 1.00 for the decline of platelets and 0.19 for the time to PSA progression. The *p* values for the second therapy were as follows: 0.74 for best PSA response; 0.17 for decline of leucocytes, 0.23 for the decline of platelets and 0.61 for the time to PSA progression. The statistical analysis could not be applied in the group with three therapies due to low number of patients (*n* = 3).

Xerostomias higher than grade 1 occurred more frequently in patients with a higher number of therapies (Table 4). The duration evaluation of this side effect was challenging. Most patients reported recovery from xerostomia after a few weeks. The duration appeared to be longer after the second or third therapy in most cases.

Dysgeusia occurred only in one patient after the second therapy (Table 4). Xerophthalmia was observed only in one patient after the second therapy. Fatigue syndromes were rare; no higher prevalence was observed with therapy numbers.

The first therapy had the strongest effect on bone pain: the majority of the patients who presented with bone pain reported a relevant reduction of the pain (reduction of analgesics or improved well-being) after the therapy. The second and third therapy led significantly less frequently to pain reduction (Table 4).

### Overall survival

The median overall survival (mOS) of the patients after the first therapy with ^131^I-MIP-1095 was 17 months (Supplementary Table 1). At the submission of this manuscript, four patients were still alive; all of these four patients received abiraterone or enzalutamide after biochemical relapse following ^131^I-MIP-1095 therapies. The time to PSA progression of these patients was for patient 3: 64 days after the first therapy and progressive from the beginning of the second therapy; for patient 4: 649 days after the first therapy; for patient 31: 85 days after the first therapy and for patient 33: 735 days after the first therapy.

## Discussion

We retrospectively report the long-term follow-up of 34 patients who received ≥1 treatment with ^131^I-MIP1095 PSMA RLT between 2011 and 2013.

Until now—and regardless of the used radiopharmaceutical—no prospective clinical trials about PSMA RLT were initiated by pharmaceutical companies. Therefore, all experience with PSMA RLT is based on salvage therapies that were administered as an “unproven intervention in clinical practice” (Helsinki Declaration). A pre-requisite for this approach is that patients previously exhausted (or were considered “unfit” to receive) all approved therapies. Thus, in contrast to a clinical trial, inclusion criteria are not constant over time. In regard to CRPC, the spectrum of approved pharmaceuticals changed dramatically during the reported inclusion period.

In Europe, abiraterone was approved for patients pre-treated with docetaxel in 09/2011 and before chemotherapy in 01/2013. Enzalutamide was first approved 06/2013 to be used after docetaxel and in 12/2014 to be used before docetaxel. Cabazitaxel is approved as a second-line chemotherapy only, since 2011. In 12/2012 ^223^RaCl was approved for a subgroup of patients with bone-confined tumor spread. Dependent on the actual approved indication, practical availability and the patient’s appropriateness to receive chemotherapy, which was classified by the responsible uro-/oncologist independently from the nuclear medicine physician, alternative options were given priority even if patients presented with a good response to the previous PSMA RLT. This explains the low number of 2nd and 3rd therapies, despite >70% of patients having a decline of >50% in serum PSA and an mTTP of 116 days after the first treatment.

Nevertheless, despite being heterogeneous, these data are still of particular interest because they present the only experience with PSMA RLT in the pre-abiraterone, pre-enzalutamid, pre-^223^RaCl era. In contrast, reports in recent publications about ^177^Lu-labeled PSMA RLT have been for patients after secondary hormone manipulation [13, 14, 20, 23–25]. There is growing experience about cross-resistance between abiraterone and enzalutamide [26] as both drugs are targeting the androgen receptor (AR) axis. If it would be possible to keep these treatment lines apart by interleaving non-AR-targeting therapies such as PSMA RLT, the interruption of selection pressure eventually might reverse cross-resistance. One case report about restored hormone response after ^177^Lu-PSMA-617 generates a promising thesis [27]. In our collective, there are four still-living patients, which were successfully bridged by ^131^I-MIP1095 RLTs until novel options became available and who now present with enduring responses to the succeeding secondary hormone manipulations.

In contrast to the before-mentioned publications about PSMA RLT [13, 14, 20, 23–25], in our cohort, the succeeding application of the radiopharmaceutical was delayed until progression of disease. One observation is that the mTTP after each further treatment decreases. Treatment regimens administered in cycles of fixed intervals shorter than the here-observed mTTP of 75 days (2.5 months) might be one option to prolong progression-free survival. In 2013, our department switched to PSMA-RLT intervals of every two months and the mTTP doubled to about 5 months [14] which is also in accordance to the prolonged PFS reported by other groups [13, 20]. Nevertheless, it remains unclear whether a prolonged mPFS or mTTP achieved by intensified treatment regimens will finally also translate into a longer mOS.

Based on the dosimetry estimates with ^124^I-MIP1095 PET and extrapolation to ^131^I-MIP1095 RLT [22], the mean absorbed doses to salivary glands are 4.62 Gy/GBq and to the red marrow 0.31 Gy/GBq. A therapy with 4 GBq ^131^I-MIP1095, therefore, corresponds to an 18.5-Gy salivary gland dose and a 1.2-Gy red marrow dose. A red marrow dose of 2 Gy is considered safe for RLT and, therefore, the here-reported low number of grade 3 hematological toxicities (6%) after the first therapy are within the expectations. Nevertheless, as demonstrated by Fig. 4, a moderate depression of blood cell count was observed after each cycle with the nadir around 6 weeks post-therapy. PSMA RLT with the radiolabeled antibody ^177^Lu-J591 resulted in comparable red marrow doses [9] and hematological toxicity as well as the platelet nadir are also in accordance with our results [28]. In contrast, with ^177^Lu-PSMA-617, the red marrow dose is about 0.025–0.03 Gy/GBq [14, 29] and therapy with 6 GBq ^177^Lu-PSMA-617 translates into an estimated red marrow dose of 0.15–0.2 Gy. Thus, the even better tolerability of this radioconjugate regarding hematological toxicity is reasonable.

With ^177^Lu-PSMA-617, 1.4 Gy/GBq (e.g. 8.4 Gy for 6 GBq) has been calculated as the salivary gland’s absorbed dose [29]. Thus, it is plausible that we find a higher incidence of moderate xerostomia with our treatment regimen than it is reported for ^177^Lu-PSMA RLT [13, 14, 20, 23–25]. However, with ^131^I-MIP-1095, we also found a higher rate of responders in regard to both “any PSA decline” (94%) and “>50% PSA decline” (71%). In the largest report (*n* = 82) about ^177^Lu-PSMA-617 administered in fractions of 6 GBq [30], the corresponding response rates for “any PSA decline” (64%) and “>50% PSA decline” (31%) were remarkably lower. As the variability of absorbed dose to different tumor lesions is high, no reliable comparison between the two ligands regard to their therapeutic range is possible. We would emphasize that dose escalation of ^177^Lu-PSMA-617 will increase both response rate and toxicity. Alternatively, de-escalation of ^131^I-MIP-1095 seems also a reasonable concept because there was no relevant difference in response rate between patients that received <3.5 or >5 GBq ^131^I-MIP-1095 (Table 3). As stated above, the analysis of this paper was done during the pre-abiraterone and pre-enzalutamide era which is in contrast to the newer reports with ^177^Lu-PSMA-617. Clinical studies revealed an increase of PSMA expression in tissue specimens after androgen-deprivation therapy [31, 32]. At present, no data are available concerning a further increase or a decrease of PSMA expression after abiraterone and/or enzalutamide. Therefore, application of ^131^I-MIP-1095 in a similar clinical setting may deliver a better data set for comparison. On the other hand, it is known that the later a therapy line is applied, the lower the efficacy due to an increase of tumor aggressiveness [33, 34].

One reason that more centers currently rely on ^177^Lu-labeled PSMA ligands is the lower co-emission of gamma radiation. ^131^I has an 82% abundance probability for high-energetic (364 keV) γ-radiation and an 89% abundance probability for β-radiation (606 keV). In contrast, ^177^Lu has a γ co-emission of only 11% (210 keV) but a 100% abundance probability for β-radiation (490 keV). Depending on radiation protection acts, this can translate into different needs for isolation. Our patients who were treated with ^131^I-MIP-1095 had an average hospitalization time of 7 days while those treated with ^177^Lu-PSMA-617 were hospitalized for 2 days. A second reason might be the level of efforts for radiolabeling. Chelator-containing ligands can be labeled with high and robust labeling yields, the synthesis of ^131^I-MIP-1095 on-site were one reason for the high variance of treatment activity. However, this might easily be overcome by a routine production by commercial companies.

## Conclusion

The first clinical experience with therapeutic small-molecule PSMA ligands was using ^131^I-MIP-1095 as a single-cycle therapy. The here-reported experience of repeated applications of PSMA RLT after the next PSA relapse is still unique. The deepness of PSA response and duration of tumor control was most pronounced with the first therapy but repeated treatments were already facing resistance. Repeated full-dose therapy also causes more side effects such as hematological toxicity and xerostomia. This provides a rationale for metronomic or fractionated treatment regimens. The first experiences based on fractionated therapy with ^177^Lu-PSMA ligands have recently been published. However, it has yet to be proven which of these concepts will be superior with regard to overall survival.

## Electronic supplementary material

Below is the link to the electronic supplementary material.

Supplementary Figure 1 (GIF 11 kb)

High-resolution image (TIF 5493 kb)

Supplementary Table 1 (GIF 89 kb)

High-resolution image (TIF 982 kb)
