# Supplementary figures and images for: Repeated PSMA-targeting radioligand therapy of metastatic prostate cancer with 131I-MIP-1095
Source: Eur J Nucl Med Mol Imaging. 2017 Mar 9;44(6):950–9. doi: 10.1007/s00259-017-3665-9 (PMC5397661; doi:10.1007/s00259-017-3665-9)

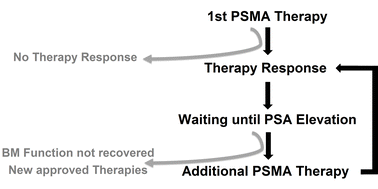

Supplement: Supplementary file 1 — (GIF 11 kb) [file 259_2017_3665_Fig5_ESM.gif]

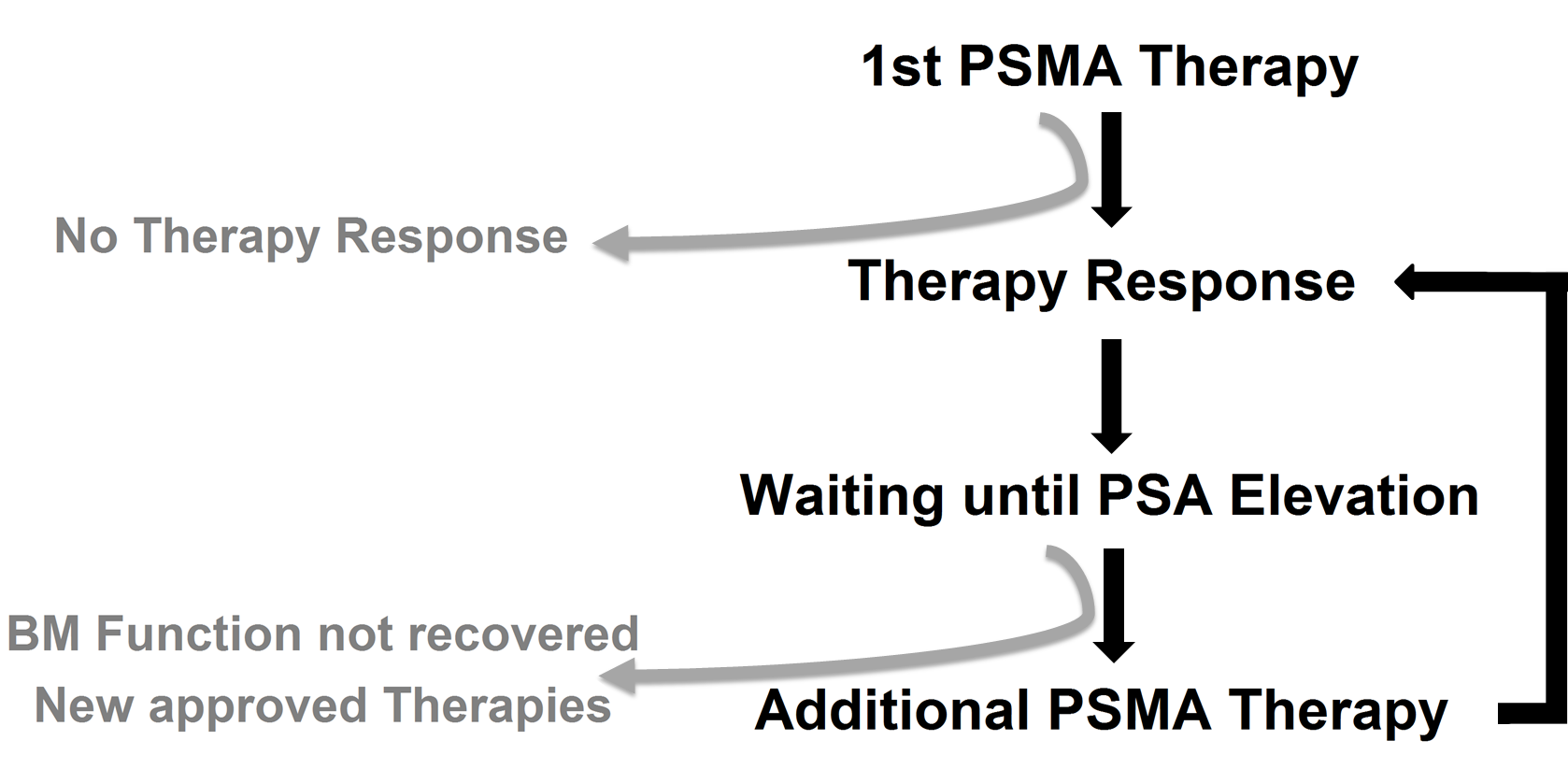

Supplement: Supplementary file 2 — High-resolution image (TIF 5493 kb) [file 259_2017_3665_MOESM1_ESM.tif]

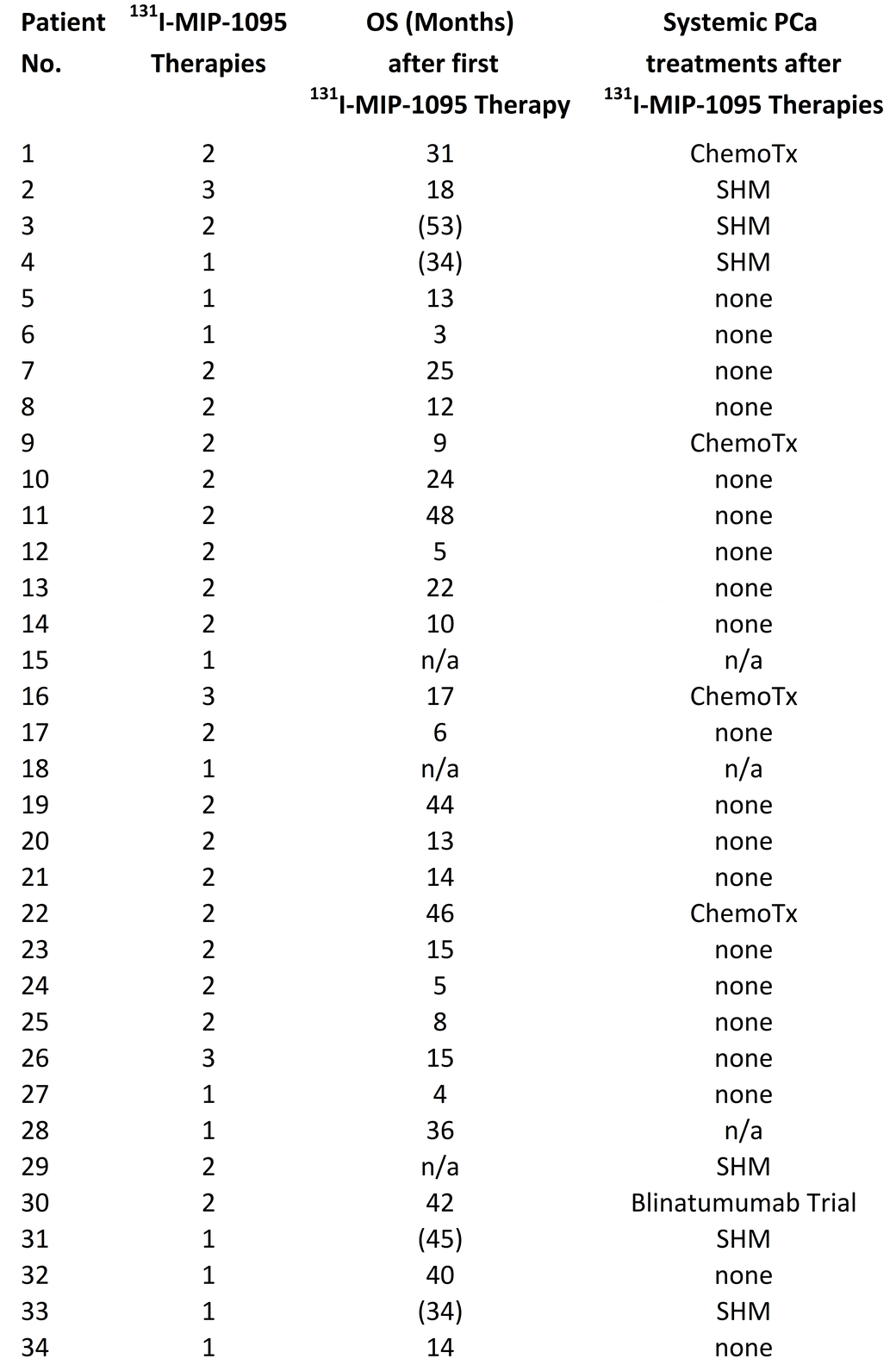

Supplement: Supplementary file 3 — (GIF 89 kb) [file 259_2017_3665_Fig6_ESM.gif]

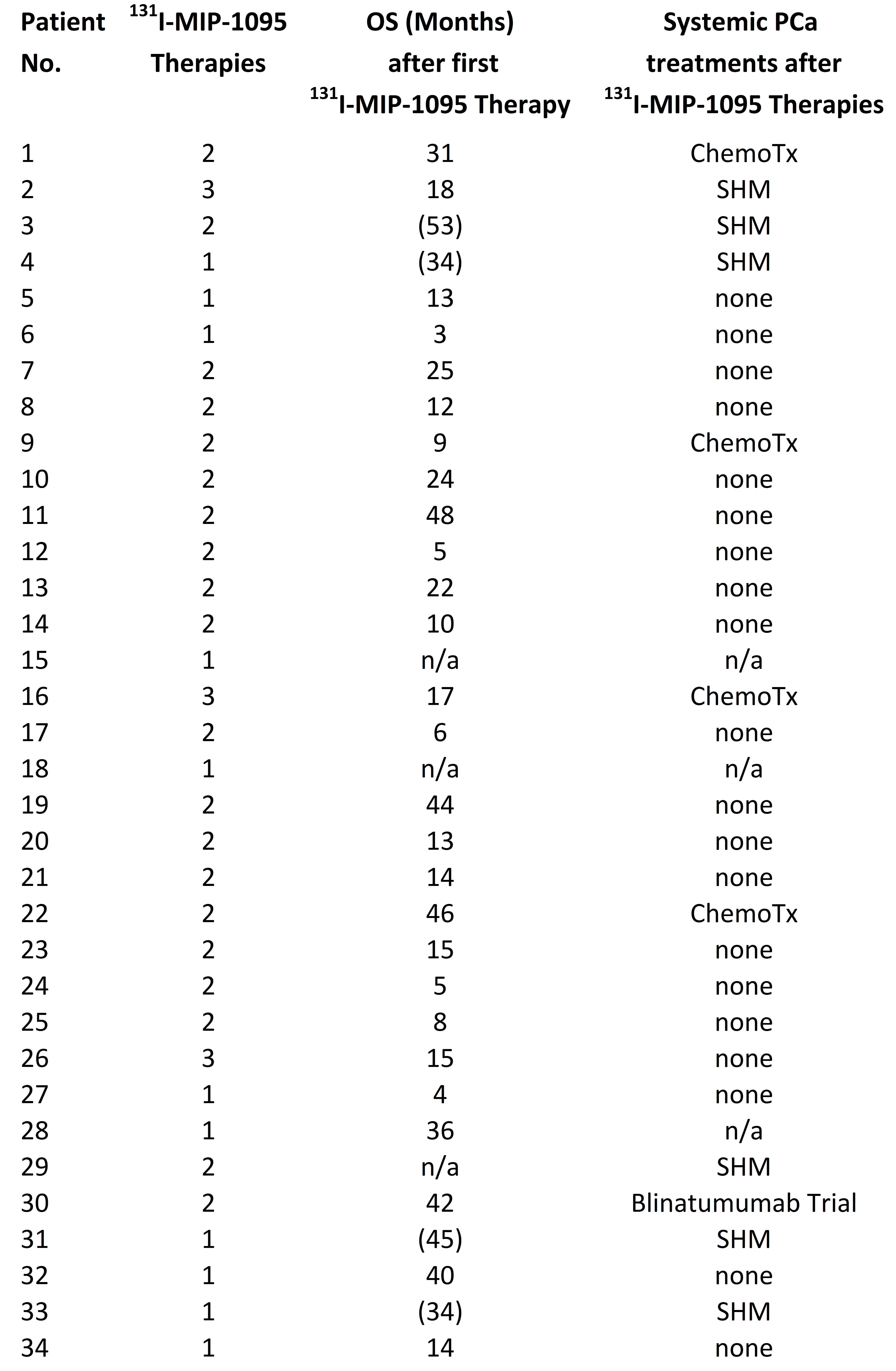

Supplement: Supplementary file 4 — High-resolution image (TIF 982 kb) [file 259_2017_3665_MOESM2_ESM.tif]
